# Comparison of the Performance of ICP-MS, CV-ICP-OES, and TDA AAS in Determining Mercury in Marine Sediment Samples

**DOI:** 10.1021/acsomega.4c06144

**Published:** 2024-11-28

**Authors:** Carolina S. Provete, Bruna M. Dalfior, Rafael Mantovaneli, Maria Tereza W. D. Carneiro, Geisamanda P. Brandão

**Affiliations:** Laboratory of Atomic Spectrometry (LEA), Chemistry Department, Federal University of Espírito Santo, Vitória, Espírito Santo 29075-910, Brazil

## Abstract

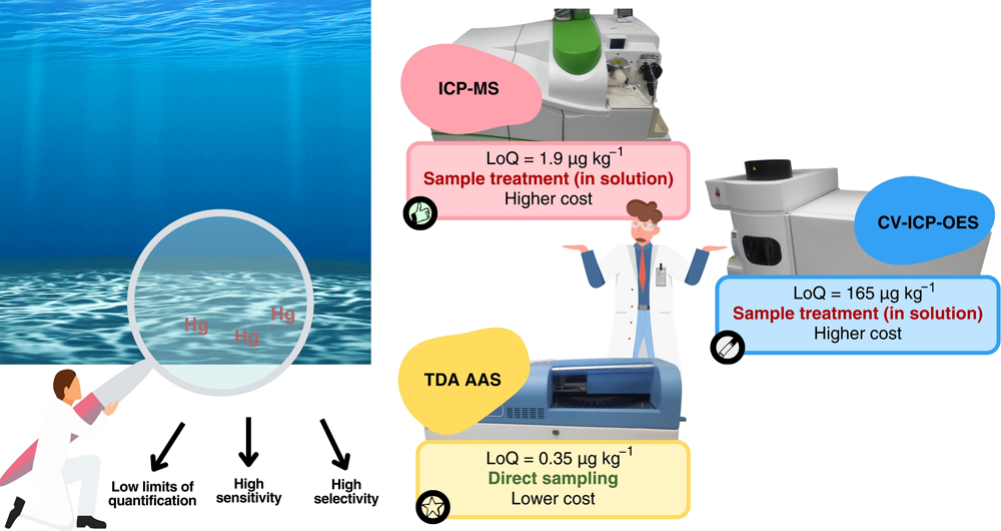

Mercury (Hg) determination in marine sediment is an analytical challenge due to the toxicity of this element even at low concentrations (up to 130 μg kg^–1^ in marine sediments) and complex matrices. Therefore, it is necessary to use analytical techniques that have high sensitivity, selectivity, and low limits of quantification (LoQ). In this study, two methods that require sample treatment and one method with direct sampling were studied. The techniques studied were inductively coupled plasma mass spectrometry (ICP-MS), inductively coupled plasma optical emission spectrometry with cold vapor generation (CV-ICP-OES), and atomic absorption spectrometry with thermodecomposition and amalgamation (TDA AAS) for Hg determination in marine sediment samples. Since ICP-MS has more studies in the literature, optimization with design of experiments was developed for CV-ICP-OES and TDA AAS. Although it was found to have low levels of instrumental LoQ for all three techniques, differences were found once the method LoQ was calculated. The calculation for method LoQ considers all analytical procedures executed, including sample treatment, which provides a 100-fold dilution for ICP-MS and CV-ICP-OES. The method LoQ obtained were 1.9, 165, and 0.35 μg kg^–1^ for ICP-MS, CV-ICP-OES, and TDA AAS, respectively. Comparing marine sediment sample analyses, Hg concentrations had no statistical difference when determined by ICP-MS and TDA AAS. It was not possible to determine Hg in marine sediment samples by CV-ICP-OES due to the high method LoQ obtained (165 μg kg^–1^). Although ICP-MS has the advantage of being a multielemental technique, it is high-value equipment and needs a large volume of argon, which has a high cost in the market, and it requires sample treatment. On the other hand, TDA AAS-based spectrometer DMA-80 performs direct sampling, avoiding the pretreatment stage, and has a relatively lower cost, both in terms of initial investment and maintenance, while maintaining the high sensitivity, accuracy, and precision required for Hg determination on marine sediment samples.

## Introduction

Mercury (Hg) is an element of environmental interest due to its high toxic potential, even at low concentrations (up to 130 μg kg^–1^ of total Hg in marine sediments).^1,2^ Hg can cause damage to the nervous, motor, renal, cardiovascular, immune, and reproductive systems of animals and other living beings.^3−5^ The forms of mercury found in nature (organic, inorganic, and elemental) have different pathways and toxicity to living organisms. Methylmercury (MeHg) and inorganic mercury (IHg) are highly bioaccumulated and biomagnified at the trophic levels. However, MeHg can be considered more hazardous because it has a slower excretion rate than IHg (70–80 days for MeHg and 30–60 days for IHg).^6−9^ Indiscriminate anthropogenic use of mercury led to a high incidence of environmental accidents involving Hg in the 19th and 20th centuries.^5,10−13^ The case that brought notoriety to the adverse effects that mercury causes on human beings occurred in Minamata Bay, Japan, in the 1950s and 1960s.^14^ The long-term damage caused in the case of Minamata Bay was related to the association of mercury with sediment particles. Sediment could act as a reservoir of elements, which may provide the (re)availability of mercury and increase bioaccumulation and biomagnification in the marine faune.^5,13,15,16^ Studies on uncontaminated areas show values of Hg in the order of 10 μg kg^–1^ or lower than 130 μg kg^–1^, that is, the threshold effects level (TEL) defined as the upper limit of contaminant concentrations in sediments without adverse effects on the biota.^17,18^ For contaminated areas, contamination of Hg in sediments can vary from TEL up to values as high as 19,800 μg kg^–1^.^17−19^

Thus, to verify the level of contamination in marine sediment samples with Hg, techniques with high sensitivity and selectivity are needed, achieving good limits of quantification (LoQ).^3,7^ Atomic spectrometry techniques are widely used because they are sensitive, selective, and often specific. It can be highlighted the techniques of inductively coupled plasma mass spectrometry (ICP-MS),^20−25^ inductively coupled plasma optical emission spectrometry with cold vapor generation (CV-ICP-OES),^26−28^ cold vapor generation atomic absorption spectrometry (CV AAS),^29−33^ and atomic absorption spectrometry with thermodecomposition and amalgamation (TDA AAS), where TDA AAS is the principle of the direct mercury analyzer (DMA-80) spectrometer by Milestone.^34−40^

Some of those techniques can be used for more detailed analyses, such as speciation or fractionation. Speciation analysis of mercury is important because of the different toxicities of organic and inorganic Hg. Atomic spectrometry can be coupled with chromatographic techniques for the determination of different species of Hg, such as HPLC-ICP-MS.^41,42^ Usually, those analyses require high analytical standards and control, and it involves a lot of steps for sample treatment.^43^ Studies have found difficulties in distinguishing between MeHg and IHg in marine sediment samples since the organic form is found in smaller concentrations.^41,43^

As an alternative for those techniques, studies have been conducted for Hg fractionation with TDA AAS using the DMA-80.^44−46^ The studies vary the heating temperature and have differentiated Hg forms: (i) at 125 °C, it is found Hg_gaseous_; (ii) at 175 °C, labile Hg (HgCl_2_, HgBr_2_, HgI_2_, and Hg(CN)_2_); at 225 °C, MeHg is released, and it is found Hg associated with humic substances and strong complexes ((CH_3_COO)_2_Hg, Hg(NO_3_)_2_·H_2_O, Hg(SCN)_2_, and Hg(ClO_4_)_2_·*x*H_2_O); at 325 °C, insoluble HgS; at 475 °C, semiliquid compounds (HgO, HgSO_4_, and HgF_2_); and at 750 °C, the residual Hg.^44,46^ Although it has problems associated with this type of analysis, such as quick consumption of the heater,^44^ with this technique it is not required sample treatment, which turns the technique “greener”^47^ and reduces sources of interferences.

Even though it is important to understand the forms in which Hg occurs in nature, marine sediment guidelines often determine limits according to the concentration of total mercury (THg). Aiming at compliance with marine sediment quality guidelines, the focus of this study was on THg determination on marine sediment samples. For that matrix, good analytical figures of merit and low levels of LoQ can be found for ICP-MS (3.1–100 μg kg^–1^),^20−22,25^ CV-ICP-OES (56.7–360 μg kg^–1^),^26−28^ and TDA AAS using the DMA-80 (0.17 to 20 μg kg^–1^).^34−36,39^ The lowest levels of LoQ for the DMA-80 can be acquired since this spectrometer is specific for Hg and has an automated direct sampler. Hence, it does not require a preparation step in which the sample is transformed to a solution and the analyte is diluted, differently than the ICP-MS and CV-ICP-OES.

Since marine sediment is a matrix of environmental interest and Hg has a high toxic potential, the main goal of this study was to compare the determination of Hg in marine sediment samples by ICP-MS, CV-ICP-OES, and TDA AAS. Although elemental techniques, the form of signal detection is different for all three techniques, and it should be highlighted that sample treatment and equipment costs are also different. It indicated the main characteristics, advantages, and disadvantages, in addition to evaluating the suitability of the techniques based on analytical figures of merit.^48,49^ Moreover, it was verified compliance with the lowest Hg concentration limits defined in marine sediment quality guidelines and standards from NOAA (United States of America’s National Oceanic and Atmospheric Administration), CCME (Canadian Council of Ministers of the Environment), and CONAMA (Brazil’s National Environment Council).^1,2,50^

## Methods

### Instrumentation

For the analysis by ICP-MS, the PerkinElmer (USA) model NexIon 300D spectrometer was used. A glass cyclonic nebulization chamber with a shield, seaspray concentric nebulization (PerkinElmer, USA), a quartz torch, and an interface with three nickel cones were used, and the operation conditions of the equipment are as described in Table S1 (Supporting Information). As a cleaning method for the sample introduction system during the analysis, a solution with 1 mg L^–1^ Au in 2% HNO_3_ v v^–1^ for 60 s was used.

For Hg determination by TDA AAS, the Milestone Srl (Italy) model DMA-80 Dual Cell spectrometer was used. The operating conditions are described in Table S2 (Supporting Information).

For the analysis by ICP-OES, a PerkinElmer (USA) Optima 7000DV spectrometer was used. The cold vapor (CV) generation system used was the FIAS Mercury/Hydride Chemifold from PerkinElmer (USA). Compressed air as shear gas, an alumina injector (2.0 mm), a one-slot quartz torch, and an axial view of the torch were used, and operation conditions of the equipment are as described in Table S1 (Supporting Information).

For acid decomposition, the microwave MW Multiwave GO (ANTON PAAR, Austria) was used.

### Reagents and Standard Solutions

All glassware and polypropylene tubs were decontaminated as described in “Supporting Information”.

In solutions and sample treatment procedures, ultrapure water, H_2_O_2_ 30% m m^–1^ (SIGMA-Aldrich, USA), and purified acids HNO_3_ 63% v v^–1^ (SYNTH, Brazil) and HCl 37% v v^–1^ (SYNTH, Brazil) were used by distillation with Distillacid BSB939 IV (BERGHOF, Germany). For the generation of cold vapor, SnCl_2_ (DINÂMICA QUÍMICA CONTEPORÂNEA LTDA, Brazil) properly diluted to 7% m v^–1^ in HCl 4% v v^–1^ was used.

The standard Hg solutions were prepared from a daily dilution of a 10 μg mL^–1^ monoelemental solution of Hg (PerkinElmer, USA). For the TDA AAS, the calibration curve was prepared in HCl 7% v v^–1^, with a working range of 0.1 to 10.0 ng. For the ICP-MS and CV-ICP-OES, solutions were prepared in HNO_3_ 2% v v^–1^ with a working range from 0.050 to 5.0 and 2.0 to 40 μg L^–1^, respectively. The internal standard (IS) solution was prepared from a monoelemental solution of 1000 mg L^–1^ of Ir (PLASMACAL, Canada) with a final concentration of 5 μg L^–1^ in HNO_3_ 2% v v^–1^. The cleaning solution with Au 1 mg L^–1^ in HNO_3_ 2% v v^–1^ was prepared from the dilution of a monoelemental solution of Au 1000 mg L^–1^ (PLASMACAL, Canada).

To compare the methods, a certified reference material (CRM) NIST (National Institute of Standards and Technology) 2702 Marine Sediment (NIST, USA) containing (447.4 ± 6.9) μg kg^–1^ Hg was used. It also used eight marine sediment samples that were collected in the Espírito Santo Bay in isobaths from 5.5 to 24 m of water column with the aid of a Van Veen grab.

### Sample Treatment

The samples were dried in an oven at 60 °C until a constant weight. Then, they were quartered and sieved on nylon screens with an opening of 250 μm. The subsamples obtained after quartering were divided for direct determination with TDA AAS and microwave-assisted acid decomposition for the ICP-MS and CV-ICP-OES.

The method used in the decomposition was carried out in the microwave MW Multiwave GO (ANTON PAAR, Austria), in which about 0.2500 g of the sample was weighed in microwave-safe flasks, and 2.00 mL of HNO_3_ 63% v v^–1^, 1.50 mL of H_2_O_2_ 30% m m^–1^, and 4.50 mL of ultrapure H_2_O were added.^51^ The mixture was allowed to predecompose for 15 min and then subjected to a heating program based on the U.S. EPA 3051A^52^ that consisted of a 5 min heating ramp to 180 °C, a 10 min hold at 180 °C, and cooling. The solutions containing the samples were quantitatively transferred to polypropylene flasks and made up to 25.00 mL with ultrapure water.^51,53^

All determinations were performed in triplicate.

### Design of Experiments for Hg Determination by CV-ICP-OES

To verify the cold vapor generation system, a full factorial design 2^3^ with a central point (CP) was carried out. The optimized parameters were reductant (SnCl_2_) concentration (Rd), acid (HCl) concentration (Ac), and sample aspiration rate (Tx). Table 1 shows the levels of the parameters with the 253 nm (I) and 194 nm (II) spectral lines.

**Table 1:** Full Factorial Design 2^3^ with a Central Point (CP) Experimental of the Optimized Parameters of CV-ICP-OES: Reductant (SnCl_2_) Concentration (Rd), Acid (HCl) Concentration (Ac), and Sample Aspiration Rate (Tx)

experiments	Rd (% m v^–1^)^a^	Ac (% v v^–1^)^a^	Tx (mL min^–1^)^a^	analytical signal intensity obtained	
				253 nm	194 nm
1	1.00 (−1)	3.00 (−1)	0.50 (−1)	141	53
2	12.00 (+1)	3.00 (−1)	0.50 (−1)	107	50
3	1.00 (−1)	10.00 (+1)	0.50 (−1)	123	58
4	12.00 (+1)	10.00 (+1)	0.50 (−1)	156	67
5	1.00 (−1)	3.00 (−1)	3.00 (+1)	790	382
6	12.00 (+1)	3.00 (−1)	3.00 (+1)	696	367
7	1.00 (−1)	10.00 (+1)	3.00 (+1)	641	330
8	12.00 (+1)	10.00 (+1)	3.00 (+1)	725	384
9 (CP)	6.50 (0)	6.50 (0)	1.75 (0)	521	241
10 (CP)	6.50 (0)	6.50 (0)	1.75 (0)	478	229
11 (CP)	6.50 (0)	6.50 (0)	1.75 (0)	442	250

a Numbers in parentheses are coded data of the experiments.

Considering the few numbers of studies using CV-ICP-OES, the values of the parameters Rd and Ac were established according to the literature for Hg determination in soil, sediment, and marine sediment samples by CV-ICP-MS and CV-ICP-OES.^28,54,55^

The analytical signal was obtained by adding a concentration of 5 μg L^–1^ Hg to a sample of decomposed marine sediment at a low concentration. For each experiment, the analysis of the corresponding analytical blank was performed, and the analytical signal obtained was subtracted from this blank in order to calculate the effects of the parameters. The planning result was calculated using an Excel spreadsheet developed by Teófilo and Ferreira.^56^

The optimized values for Hg determination in marine sediment samples by CV-ICP-OES were determined as 7% m v^–1^ SnCl_2_ solution in 4% v v^–1^ HCl, sample aspiration rate of 4 mL min^–1^, and ionic spectral line of 194 nm.

### Design of Experiments for Hg Determination by TDA AAS

To verify the TDA AAS system, a fractional factorial design 2_*V*_^5–1^ was applied with the following variables: sample mass (*m*), drying/decomposition heating ramp (*t*_a_), decomposition temperature (*T*), hold decomposition time (*t*_d_), and purge time from the furnace to the amalgamator (*t*_p_).

The design was assembled with contrast 5 = 1234, and results from the experiments were obtained according to eq 1 (Table 2).
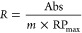
1Abs is the absorbance subtracted from the blank, *m* is the sample mass, and RP_max_ is the greater response value obtained through . Effects values were calculated with an Excel spreadsheet developed by Teófilo and Ferreira.^56^

**Table 2:** Fractional Factorial Design 2_*V*_^5–1^ for Variables Trial of TDA AAS System with DMA-80: Sample Mass (*m*), Drying/Decomposition Heating Ramp (*t*_a_), Decomposition Temperature (T), Hold Decomposition Time (*t*_d_), and Purge Time from the Furnace to the Amalgamator (*t*_p_)

experiments	*m* (mg)^a^	*t*_a_ (s)^a^	*T* (°C)^a^	*t*_d_ (s)^a^	*t*_p_ (s)^a^	*R* obtained^a^
1	50 (−1)	90 (−1)	600 (−1)	30 (−1)	90 (+1)	0.80
2	100 (+1)	90 (−1)	600 (−1)	30 (−1)	30 (−1)	0.81
3	50 (−1)	150 (+1)	600 (−1)	30 (−1)	30 (−1)	0.81
4	100 (+1)	150 (+1)	600 (−1)	30 (−1)	90 (+1)	0.80
5	50 (−1)	90 (−1)	750 (+1)	30 (−1)	30 (−1)	0.86
6	100 (+1)	90 (−1)	750 (+1)	30 (−1)	90 (+1)	0.90
7	50 (−1)	150 (+1)	750 (+1)	30 (−1)	90 (+1)	0.92
8	100 (+1)	150 (+1)	750 (+1)	30 (−1)	30 (−1)	0.96
9	50 (−1)	90 (−1)	600 (−1)	90 (+1)	30 (−1)	0.80
10	100 (+1)	90 (−1)	600 (−1)	90 (+1)	90 (+1)	0.85
11	50 (−1)	150 (+1)	600 (−1)	90 (+1)	90 (+1)	0.82
12	100 (+1)	150 (+1)	600 (−1)	90 (+1)	30 (−1)	0.85
13	50 (−1)	90 (−1)	750 (+1)	90 (+1)	90 (+1)	0.93
14	100 (+1)	90 (−1)	750 (+1)	90 (+1)	30 (−1)	0.98
15	50 (−1)	150 (+1)	750 (+1)	90 (+1)	30 (−1)	0.97
16	100 (+1)	150 (+1)	750 (+1)	90 (+1)	90 (+1)	0.96

a Numbers in parentheses refer to coded experiment data. *R* is normalized results (eq 1).

The optimized values for Hg determination in marine sediment samples by TDA AAS were determined as 100 mg of sample mass (*m*), 120 s for drying/decomposition heating ramp (*t*_a_), 650 °C of decomposition temperature (*T*), 60 s for hold decomposition time (*t*_d_), and purge time from the furnace to the amalgamator (*t*_p_).

### Analytical Figures of Merit

The working ranges and analytical figures of merit that were evaluated: linearity (determination coefficient, *R*^2^), sensitivity, limit of detection (LoD), limit of quantification (LoQ), precision (repeatability), and accuracy (analyte recovery and CRM analysis). The instrumental LoD and LoQ were calculated using the simplified method of estimation from the calibration curve (eqs 2 and 3).^48^
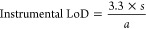
2
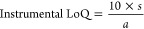
3where *s* is the standard deviation of 10 replicates of the blank decomposition solution and *a* is the sensitivity of the analysis determined by the linear regression of the analytical curve.

For the calculation of LoQ in μg kg^–1^ (method LoQ), it is necessary to consider all analytical methods, sample treatment included. Therefore, for the direct analytical method TDA AAS, using DMA-80, the LoQ was only divided by sample mass. For the other two nondirect strategies that require sample treatment, ICP-MS and CV-ICP-OES, the instrumental LoQ was divided by the sample mass (*m*_s_) and multiplied by the dilution volume (*V*_d_) utilized for the decomposition (eq 4).
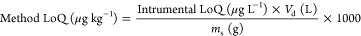
4

The comparison between the determined and certified values of the CRM by the methods studied was performed with the statistical test of the Institute for Reference Materials and Measurements (IRMM) (eq 5).^57^
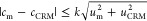
5where *c*_m_ is the concentration determined, *c*_CRM_ is the concentration certified in the CRM, *k* is the coverage factor given by the certificate of the CRM, *u*_m_^2^ is the uncertainty (standard deviation) obtained with the determination, and *u*_CRM_^2^ is the uncertainty certified in the CRM. If the condition given by the equation is met, then there is no statistical difference with 95% confidence between the determined and certified values. If the condition is not met, then there is a statistical difference between the determined and the certificate values.

The statistical comparison of the determined values of Hg in marine sediment samples among the methods was carried out with the use of the *t*-Student test with 95% confidence (eq 6).
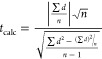
6where *d* is the difference between two results from different techniques and *n* is the number of replicates. If the *t*_calc_ value was lower than the *t*_tab_ value with a significance level of 0.05, then it was considered that there was no statistical difference between the two determined values.

## Results and Discussion

When comparing the number of studies and the information provided for the determination of Hg in marine sediments, it was observed that most studies used the ICP-MS technique, some used ICP-OES, and few used TDA AAS, such as DMA-80 (Figure S1 in “Supporting Information”). Therefore, for ICP-MS determinations, only a few studies were performed (Supporting Information), in addition to method verification (Table S3 in “Supporting Information”). An assessment was conducted for the cleaning conditions of the sample introduction system (cleaning time and cleaning solution composition) to minimize the possible memory effect as well as the analytical figures of merit calibration curves when using different internal standards (IS). Optimized conditions are described in Instrumentation and in “Supporting Information”.

In the case of CV-ICP-OES and TDA AAS, due to the lower availability of studies for determining Hg in marine sediment samples, it was sought to optimize the most relevant parameters for these techniques.

### Design of Experiments for Hg Determination by CV-ICP-OES

To better understand the interaction among the analytical signal (main line of 253 nm (I) and secondary line of 194 nm (II)) and the parameters of the cold vapor system, a full factorial design 2^3^ with a central point (Table 1) was conducted with the parameters: SnCl_2_ reductant concentration (Rd), HCl concentration (Ac), and sample aspiration rate (Tx). To evaluate the results of Table 1, the analysis of variance (ANOVA) was performed (Table S4 in “Supporting Information”). With ANOVA, it was verified that the mathematical model obtained had significant regression since the *F*_calc_ for the 253 nm (28) and 194 nm (41) lines were greater than the *F*_tab_ (8.9). Moreover, it has no lack of fit since the *F*_calc_ for the 253 nm (4.7) and 194 nm (15) lines were less than the *F*_tab_ (19).

With the adequacy of the elaborated model verified, it was calculated the effects and effects errors of the experiments (Table S5 in “Supporting Information”).

The results indicated that the effects of Rd and Ac were not statistically significant. To ensure that the analytical signal responses were within the experimental domain, Rd was set at a value close to the central point at 7% m v^–1^. On the other hand, Ac was set at 4% v v^–1^, aiming to keep the acidity low, both to avoid greater wear of the ICP-OES consumables and to have a lower consumption of reagents. As only Tx has a significant effect on the system and the effect value was positive (indicating that the higher the value, the greater the analytical signal), Tx of 3 and 4 mL min^–1^ were used in this optimization. Higher aspiration rates were not possible to work with due to the limitations of the cold vapor generation system used.

Table 3 presents the analytical figures of merit for the rates of 3 and 4 mL min^–1^ using lines 253 and 194 nm. There were good determination coefficients (*R*^2^) of the calibration curves, above 0.99, for all conditions studied. However, relatively high method LoQ were observed when compared to Hg concentrations in marine sediment samples for uncontaminated environments.^1,2^

**Table 3:** Analytical Figures of Merit Obtained for Hg Determination by CV-ICP-OES Using a Sample Aspiration Rate (Tx) of 3 and 4 mL min^–1^ in Spectral Lines 253 and 194 nm

sample aspiration rate (mL min^–1^)	3		4	
spectral line (nm)	253	194	253	194
sensitivity (intensity L μg^–1^)	173	85	181	87
linear coefficient (μg L^–1^)	513	4.5	400	–43
determination coefficient (*R*^2^)	0.9996	0.9997	0.9985	0.9976
instrumental LoD (μg L^–1^)	0.77	0.71	0.73	0.54
instrumental LoQ (μg L^–1^)	2.3	2.2	2.2	1.6
method LoQ (μg kg^–1^)	232	218	222	165
determined concentration of CRM NIST 2702^a^ (μg kg^–1^)	<232	388 ± 99 (26%)	<222	444 ± 101 (23%)

a Certified value (447.4 ± 6.9) μg kg^–1^. Values in parentheses refer to RSD.

Although the determined method LoQ was high with these operating conditions, it is important to note that the cold vapor generation technique was able to reduce by about 100 times the limit in relation to the method LoQ reported in the literature for the determination of Hg in sediments (marine, river and brackish water) and soil by ICP-OES.^58−62^ If the analyst is interested, the LoQ could be improved, for example, by using a preconcentration step of the mercury vapor with a gold amalgam. The study conducted by Hellings, Adeloju, and Verheyen^55^ achieved a method LoQ of 10 μg kg^–1^ in plant and soil samples with concentrations of SnCl_2_ and HCl with the values of 20% m v^–1^ and 17% v v^–1^, respectively, even though the experimental domain of the Full Factorial Design used in this study has shown that higher levels of Rd and Ac have a low impact on the analytical signal (being set at 7% m v^–1^ for Rd and 4% v v^–1^ for Ac). However, considering that in the CV generation system used have an aspiration rate of Rd and Ac twice the sample aspiration rate (6 and 8 mL min^–1^ for studied levels), large volumes of waste are generated with high concentrations of tin (potentially toxic waste). Therefore, it was decided not to work with higher concentrations to be in line with the principles of Green Analytical Chemistry.^47,63^

There was no statistical difference between the sensitivities for the calibration curves (Table 3) with the same spectral line. In the 194 nm line, a great variability of the data in the determination of Hg was observed in the sample of CRM NIST 2702. The relative standard deviation (RSD) for this line was 26 and 23%, with the rates of 3 and 4 mL min^–1^, respectively.

For the 253 nm line, it was not possible to determine the Hg concentration in the CRM, considering that the concentration determined was below the method limit of quantification. The hypothesis for this is detailed in the “Supporting Information”. As for the 194 nm line, no statistical difference was found between the determined and certified values of the CRM. Although this line has a lower sensitivity, the baseline is also more uniform (Figures S2–S5 in “Supporting Information”), providing more adequate results for the Hg determination in marine sediment samples by CV-ICP-OES.

The accuracy was also evaluated by the analyte addition and recovery test, in which Hg additions of 1 and 5 mg kg^–1^ were made (in the decomposed solution they are equivalent to 2.5 and 10 μg L^–1^). According to AOAC,^49^ recovery adequacy (range 95–105%) was only verified for the 194 nm line with Tx of 4 mL min^–1^ (Table S6 in “Supporting Information”), corroborating the results obtained in the CRM evaluation.

The evaluation of the CV-ICP-OES method accuracy was carried out by verifying the RSD obtained for the replicates of the CRM. For the aspiration rate of 4 mL min^–1^ when using the 194 nm line, the RSD in the CRM was equal to 23%. For this concentration range, the appropriate RSD would be less than 15% according to AOAC.^49^ The low precision may have occurred due to the limitation of the radiofrequency voltage of the equipment at 1000 W. The low radiofrequency can cause instability in the plasma, mainly associated with the high flow of the peristaltic pump. Low precision also directly influences the determination of method LoQ, as can be seen from the high relative value obtained, 165 μg kg^–1^.^64^

Thus, with the results shown above, the optimized conditions for the CV-ICP-OES for Hg determination in marine sediment samples were with the reductant SnCl_2_ concentration equal to 7% m v^–1^, the acid HCl concentration of 4% v v^–1^, the sample aspiration rate of 4 mL min^–1^, and an ionic spectral line of 194 nm.

### Design of Experiments for Hg Determination by TDA AAS

For the direct Hg determinations using the TDA AAS, a fractional factorial design 2_*V*_^5–1^ was carried out to screen the variables (i) sample mass (m), (ii) drying/decomposition heating ramp (*t*_a_), (iii) decomposition temperature (*T*), (iv) hold decomposition time (*t*_d_), and (v) purge time from the furnace to the amalgamator (*t*_p_). With the results of the fractional factorial design (Table 2), the contrast error of the studied variables calculated with an Excel spreadsheet^56^ (Table 4) was obtained.

**Table 4:** Values and Significance of Estimated Contrasts and Values of Contrasts Error for the Fractional Factorial Design **2**_*V*_^5*–*1^ for Direct Hg Determination Using TDA AAS

variable	estimated contrast	contrast error	significance^a^^,^^b^
*m*	0.02219	0.0091	SG
*t*_a_	0.01969	0.0091	SG
*T*	0.1187	0.0091	SG
*t*_d_	0.03769	0.0091	SG
*t*_p_	–0.007562	0.0091	NSG

a SG = statistically significant.

b NSG = statistically not significant. A significance level of 0.05.

Since the error value of the contrasts is 0.0091, only *t*_p_ (purge time from the furnace to the amalgamator) was not statistically significant. To evaluate the other studied variables, the contribution of each variable to the system was also calculated. Among the first-order contrasts, the variable with the greatest contribution was decomposition temperature (79%), followed by hold decomposition time (8.0%), sample mass (2.8%), and drying/decomposition heating ramp (2.2%).

For variable m (sample mass), the contrast was positive, indicating that the higher the mass, the better the result obtained. This result was to be expected given that sample mass is an important factor in increasing the analytical signal. However, this variable was screened in order to check whether working with lower sample masses would produce statistically different results, given that the lower the sample mass, the greater the chance of analytical errors due to homogeneity.^63^ As the result of the contrast was statistically significant, and in the tests with the lower mass, higher DPRs were obtained, it was decided to use a mass of 100 mg in order to reduce the experimental error and increase sensitivity.

Despite being statistically significant, the results of the variable contributions to the system showed that the contributions of variables *t*_a_ (drying/decomposition heating ramp) and *t*_d_ (hold decomposition time) were 2.2 and 8.0%, respectively. These values represent a low contribution when compared to the percentage of *T* (decomposition temperature), which was 79%. Therefore, only the decomposition temperature was optimized, ranging from 600 to 750 °C (Table 5). In order to avoid wear and tear on the equipment’s consumables, and following the manufacturer’s guidelines, temperatures above 750 °C were not used. The other studied variables were set after screening at *m* at 100 mg, *t*_a_ at 120 s, and t_d_ and t_p_ at 60 s. With the exception of the sample mass, the values of the variables were set at the medium of the levels of the experimental design carried out.

**Table 5:** Absorbance Results for the Decomposition Temperatures Studied

		absorbance	
experiments	decomposition temperature (°C)	medium	standard deviation
1	600	4.43	0.0040
2	650	4.63	0.13
3	700	4.67	0.21
4	750	4.96	0.051

The results obtained for the absorbance signal in the optimization were normalized by the sample mass, and it can be seen from Table 5 that there was little variation between the levels of *T* studied. There was only a statistical difference with a 95% confidence level between experiments 1 and 4.

Although the highest normalized absorbance signal was obtained in experiment 4, there was no statistical difference between this and experiment 2. Therefore, as there was no loss of sensitivity, it was decided to set the decomposition temperature at 650 °C for the optimized system to minimize wear and tear on the equipment’s consumables.

### Analytical Figures of Merit

To verify the suitability of Hg determination in marine sediment samples using the methods with ICP-MS, CV-ICP-OES, and TDA AAS, the analytical figures of merit were obtained (Table 6). The accuracy was evaluated by analyzing the certified reference material (CRM) NIST 2702 Marine Sediment, and the precision was calculated using the results of Hg determination in the marine sediment samples collected at Esprito Santo Bay.

**Table 6:** Analytical Figures of Merit of Hg Determination in Marine Sediment Samples with ICP-MS, CV-ICP-OES, and TDA AAS

analytical figures of merit	ICP-MS	CV-ICP-OES	TDA AAS^a^
working range (μg L^–1^)	0.05–5	2–40	1–100
determination coefficient (*R*^2^)	0.9984	0.9976	0.9984
instrumental LoD (μg L^–1^)	0.0079	0.54	0.11
instrumental LoQ (μg L^–1^)	0.026	1.6	0.35
method LoQ (μg kg^–1^)	1.9	165	0.35
determined value (μg kg^–1^) of CRM NIST 2702 (447.4 ± 6.9 μg kg^–1^)	430 ± 22 (5.2%)^b^	444 ± 101 (23%)^b^	462 ± 11 (2.4%)^b^
precision in sample analysis (RSD)	1.6–19%	ND^c^	2.0–16%
analytical frequency (min sample^–1^)^d^	10	10	10

a The calibration curve for TDA AAS was constructed based on the mass of Hg, but for comparison purposes, the concentration was calculated in μg L^–1^, considering that the volume used during the analysis of the standard solution was 100 μL.

b Values in parentheses referring to RSD.

c It was not possible to determine the Hg concentration in the marine sediment samples by CV-ICP-OES because they were all below the method LoQ.

d Analytical frequency calculated considering the entire analytical process (treatment and analysis).

For all techniques, good determination coefficients (>0.99) and low instrumental LoD were observed (Table 6). Considering the working range, the ICP-MS has a limitation regarding the higher concentration due to the memory effect. The time necessary for cleanup is prolonged with the use of higher concentrations of standard solutions, which consequently limits the analytical frequency of the analysis. Knowing that higher levels of standard increase the cleaning time between analyses in the ICP-MS, this would reduce the method’s analytical frequency. On the other hand, the CV-ICP-OES has its lower concentration limited due to the relatively high instrumental LoQ value determined. For TDA AAS, the possibility to work with higher concentrations of Hg was verified without impact on the analytical frequency, which is an advantage when working with samples with different scales of concentration.

All techniques showed good accuracy by evaluating the statistical agreement between the determined and the certificate values of the CRM NIST 2702. In addition, there was no statistical difference with 95% confidence among the values determined in the CRM by the three techniques. Hence, good precision was verified for both ICP-MS and TDA AAS, since the RSD values considered adequate for the range of Hg concentration found in the samples (RSD < 21%) and in the CRM (RSD < 15%) are higher than the determined values.^49^ For CV-ICP-OES, an RSD value was obtained above the one recommended by the AOAC for the CRM concentration range. Moreover, it was not possible to determine the RSD in the marine sediment samples collected in Esprito Santo Bay with the CV-ICP-OES, considering that all concentrations obtained for Hg were below the method LoQ.

Although the instrumental LoQ for ICP-MS was lower than the value for TDA AAS, this technique uses direct sampling; therefore, the sample decomposition step was not necessary. In calculating the method LoQ, it is necessary to consider all analytical methods. The sample treatment resulted in a 100-fold dilution factor for the marine sediment samples. That dilution factor was incorporated into the method LoQ of ICP-MS and CV-ICP-OES, resulting in a higher method LoQ than that observed for TDA AAS. Nevertheless, obtaining a method LoQ for the ICP-MS (1.9 μg kg^–1^) close to the value obtained for the TDA AAS (0.35 μg kg^–1^) evidence the high sensitivity of the ICP-MS for Hg determination in marine sediment samples. For ICP-MS, higher sensitivity can be achieved by using preconcentration techniques such as solid phase microextraction (SPME) with carbon nanotubes or anion exchange resins.^65,66^ This makes ICP-MS a more competitive alternative to DMA-80 based on the TDA-AAS.

As for the CV-ICP-OES, even using the technique of cold vapor generation to increase the sensitivity for the Hg determination, a method LoQ about 100 times greater than that for the other techniques was obtained. This high LoQ may be due to the limitation of the reagent aspiration rate, which limits the amount of analyte taken into the plasma. It could also be due to the sensitivity limitation of the ICP-OES, normally inferior to the methods for Hg determination by ICP-MS or TDA AAS.^58^ Another factor that may have impacted the LoQ was the limitation of the radiofrequency generator of the equipment used, which could not be operated at voltages greater than 1000 W. In addition, the ionic line of Hg (194 nm) was used, which may have had its sensitivity affected by the low power, since to ionize and excite it would be necessary to supply more energy to the analyte. However, the method LoQ value found in this study was within the range values reported for CV-ICP-OES in the literature in sediment or marine sediment samples of 56.7–360 μg kg^–1^.^26−28^

The range of method LoQ in marine sediment samples described in the literature for the ICP-MS was found to be 3.1–100 μg kg^–1^,^20−22,25^ and for the determination by TDA AAS (with DMA-80), 0.17–20 μg kg^–1^.^34−36,39^ Comparing these values with those determined in this study (ICP-MS 1.9 and TDA AAS 0.35 μg kg^–1^), it was noticed that limits of quantification that were similar to or better than those reported in the literature were obtained.

### Hg Determination in Marine Sediment Samples

After the methods were verified, the marine sediment samples collected in the Esprito Santo Bay were analyzed by the three techniques. However, all concentrations found were lower than the LoQ (165 μg kg^–1^) when using the CV-ICP-OES. Table 7 shows the concentrations of Hg determined by ICP-MS and TDA AAS in the marine sediment samples.

**Table 7:** Hg Concentration Determined in Marine Sediment Samples by ICP-MS and TDA AAS

	Hg concentration ± standard deviation (μg kg^–1^)	
samples	ICP-MS^a^	TDA AAS^a^
P1	30.9 ± 5.9 (19%)	32.52 ± 0.66 (2.0%)
P2	31.01 ± 0.51 (1.6%)	37.1 ± 1.6 (4.3%)
P3	53.2 ± 6.9 (13%)	75.8 ± 2.6 (3.5%)
P4	21.3 ± 1.2 (5.6%)	22.3 ± 1.3 (5.8%)
P5	13.2 ± 1.7 (13%)	13.1 ± 1.4 (11%)
P6	31.5 ± 1.2 (3.7%)	37.91 ± 0.80 (2.1%)
P7	45.8 ± 5.0 (11%)	60.1 ± 3.3 (5.4%)
P8	13.3 ± 1.4 (11%)	8.5 ± 1.4 (16%)

a Values in parentheses represent the relative standard deviation.

It is observed (Table 7) that there is a tendency to obtain higher concentration values when the samples were analyzed by TDA AAS. This tendency to obtain lower concentrations when using ICP-MS may be due to the need for the sample decomposition step, which can be a source of analyte loss even when performed with great analytical rigor. It could also be because of the retention of Hg vapors in the nebulization chamber and the adhesion of this element to the walls of the tubes of the sample introduction system.^67^ On the other hand, Hg determinations by TDA AAS were performed with dry samples *in natura*, minimizing possible analyte losses, and in specific equipment. Despite this, performing the *t*-Student statistical test to compare the results, it was found that the values of Hg concentration in the samples determined by ICP-MS did not show a statistical difference, with 95% confidence, in relation to those determined by TDA AAS.

Thus, comparing the three methods, if the interest of the analyst is monitoring low levels of Hg in marine sediment samples, the most appropriate ones were those that used ICP-MS and TDA AAS. The method that used the CV-ICP-OES was limited due to the aforementioned instrumental conditions, in which a high method LoQ value (165 μg kg^–1^) was obtained, a value higher than the mercury concentrations in marine sediment samples from noncontaminated regions.^1,2^

### Compliance with Sediment Quality Guides (SQG)

To determine the concentration of mercury in marine sediment samples to verify compliance with sediment quality guidelines, the methods employing the ICP-MS and the TDA AAS were adequate, even for the TEL value (130 μg kg^–1^).^1,2^ Thus, it was adequate to use these techniques for sediment quality assessment as regulated by NOAA^2^ in the USA and CCME^1^ in Canada. Using these guides as a reference, the method that used CV-ICP-OES is only adequate to assess the upper limits of TEL.

In Brazil, CONAMA Resolution No. 454 of 2012 regulates the quality of dredged marine sediment in which level 1 is defined as the “threshold below which there is less probability of adverse effects on the biota” in marine sediment samples with mercury concentration up to 300 μg kg^–1^ and as level 2 the “threshold above which there is a greater probability of adverse effects on the biota” in marine sediment samples with mercury concentration above 1000 μg kg^–1^.^50^ Thus, considering that the concentration levels described in this resolution are above the LoQ obtained in the samples for the three methods studied, including the one that used CV-ICP-OES, it would be possible to use any of them to evaluate the quality of marine sediment samples in Brazil according to Hg concentration.

## Conclusions

In general, the Hg determination methods in marine sediment samples using ICP-MS, CV-ICP-OES, and TDA AAS were adequate for the assessment of levels according to sediment quality guides. However, for the monitoring of low concentrations of Hg in marine sediment samples, only the methods that used ICP-MS and TDA AAS could be used due to the high relative method LoQ obtained (165 μg kg^–1^) when using the CV-ICP-OES. Although ICP-MS and ICP-OES have the advantage of being multielemental techniques, they require sample treatment, and they are high-value equipment and need a large volume of argon, which has a high cost in the market. On the other hand, the TDA AAS-based DMA-80 spectrometer aligns with the principles of Green Analytical Chemistry,^47^ since it is a direct sampling method and “greener” than ICP-MS and CV-ICP-OES. This is due to the fact that it avoids the sample treatment stage. Additionally, DMA-80 has a relatively lower cost, in terms of both initial investment and maintenance, while maintaining the high sensitivity, accuracy, and precision required for Hg determination on marine sediment samples.

## Supporting Information Available

Methods and results information for instrumentation and reagents (PDF)
