# Supplementary material for: Comparison of the Performance of ICP-MS, CV-ICP-OES, and TDA AAS in Determining Mercury in Marine Sediment Samples
Source: ACS Omega. 2024 Nov 28;9(50):49229–38. doi: 10.1021/acsomega.4c06144 (PMC11656208; doi:10.1021/acsomega.4c06144)
Supplement: Supplementary file 1 — ao4c06144_si_001.pdf [file ao4c06144_si_001.pdf]

Supporting Information: Methods and results, information for instrumentation and reagents.

Comparison of the performance of ICP-MS, CV-ICP-OES and TDA AAS in determining mercury in marine sediment samples

Carolina S. Provete[a], Bruna M. Dalfior[a], Rafael Mantovaneli[a§], Maria Tereza W. D. Carneiro[a], Geisamanda P. Brandão[a]*

*geisamanda@gmail.com

[a]Laboratory of Atomic Spectrometry (LEA), Chemistry Department, Federal University of Espírito Santo, Vitória, Espírito Santo 29075-910, Brazil.

Present address:
§ R.M.: Federal Institute of Espírito Santo – *campus* Alegre, Alegre, 29500-000 ES, Brazil.

Instrumentation

The operating conditions for all three techniques were acquired following the verification or optimization of the method. To ascertain the requirements for each technique (verification or optimization), a bibliometric study was conducted. The bibliometric study was performed with RStudio, and the research base comprised Web of Science and Scopus from 01/2019 to 10/2024. A total of 1672 papers in chemistry were identified through the use of the specified search terms: ("Marine Sediment" AND "Mercury") OR ("Mercury" AND "Inductively Coupled Plasma Mass Spectrometry") OR ("Mercury" AND "ICP-MS") OR ("Mercury" AND "Atomic Absorption Spectrometry with Thermodecomposition and Amalgamation") OR ("Mercury" AND "TDA AAS") OR ("Mercury" AND "Inductively Coupled Plasma Optical Emission Spectrometry") OR ("Mercury" AND "ICP-OES") OR ("Mercury" AND "Inductively Coupled Plasma Optical Emission Spectrometry with cold vapor generator") OR ("Mercury" AND "CV-ICP-OES") OR ("Marine Sediment" AND "Direct Mercury Analyser") OR ("Marine Sediment" AND "DMA-80").

The word cloud generated by RStudio revealed that the terms "DMA-80" and "cold vapor" were cited 39 and 30 times, respectively. The terms "ICP-MS," "ICP-OES," "DMA-80," and "HPLC-ICP-MS" were referenced 122, 48, 39, and 22 times, respectively. A comparison of the number of studies and the information provided for the determination of Hg in marine sediments revealed that the majority of studies employed the ICP-MS technique, while others utilized ICP-OES and a few employed TDA AAS, such as DMA-80 (Figure S1).

Therefore, for ICP-MS determinations, only a few studies were performed, in addition to method verification. In the case of CV-ICP-OES and TDA AAS, due to the lower availability of studies for determining Hg in marine sediment samples, it was sought to optimize the most relevant parameters for these techniques.

Table S1 shows operating conditions of ICP-MS and CV-ICP-OES and Table S2 shows operating conditions of DMA-80 for mercury determination in marine sediment samples.

Figure S1. Word cloud for mercury determination by spectrometric techniques.

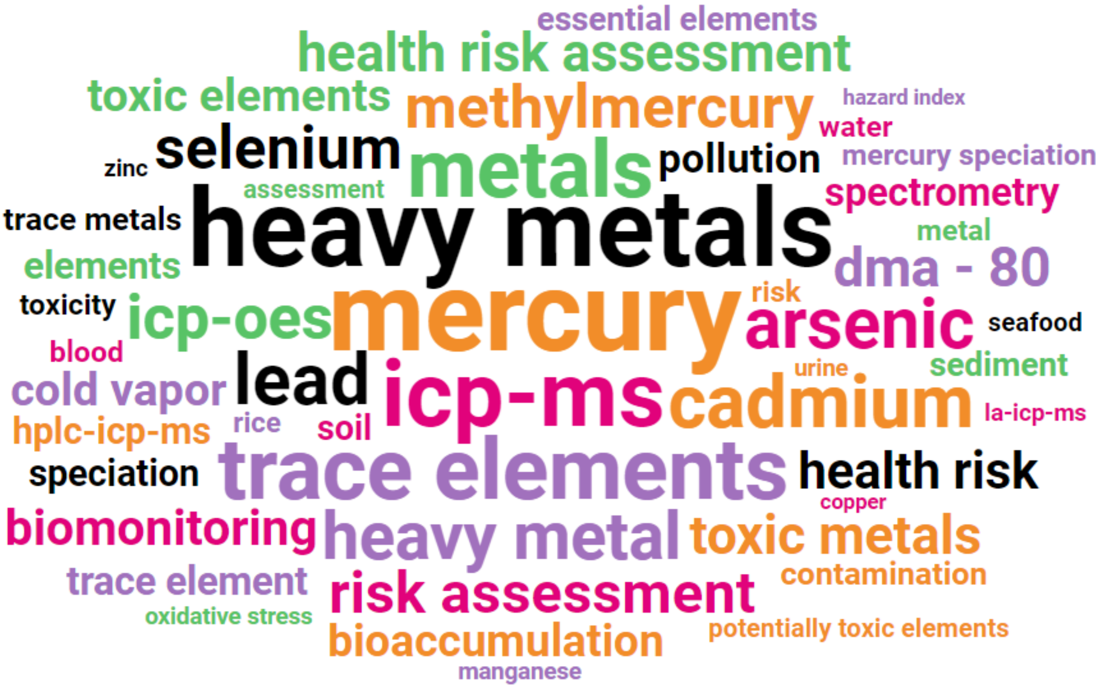

Table S1. Operating conditions of ICP-MS and CV-ICP-OES.

Parameter	ICP-MS	CV-ICP-OES
Radiofrequency power (W)	1350	1000
Plasma gas flow (L min^{-1})	16	15
Auxiliary gas flow (L min^{-1})	1.20	0.2
Nebulizing gas flow (L min^{-1})	1.1	1.5
Detection	Isotope ^{202}Hg with IS* ^{193}Ir	194 nm (ionic line) with Axial view

*IS: internal standard

Table S2. Operating conditions of TDA AAS system with DMA-80.

Parameter	DMA-80
Sample mass (g)	0.1000
Maximum start temperature (°C)	250
Drying temperature (°C)	250
Drying time (s)	60
Drying/decomposition heating ramp (s)	120
Decomposition temperature (°C)	650
Decomposition time (s)	60
Purge time (s)	60

For the generation and maintenance of plasma in the ICP-MS and CV-ICP-OES was used high purity argon of 99.9992% (AIR PRODUCTS BRASIL LTDA, Brazil). To the CV-ICP-OES primer 99.99% nitrogen gas (AIR PRODUCTS BRASIL LTDA, Brazil) was used to purge the optics and compressed air (Air Compressor SHULZ S/A, Brazil) pre-treated with dryer (HB AR COMPRIMIDO, Brazil) was used as the shear gas. Compressed air was used as carrier gas in the TDA AAS (Air Compressor DMA-80 AC, MILESTONE SRL, Italy).

Decontamination procedure

The glassware and polypropylene tubes used were washed with water and neutral detergent and were also decontaminated in an acid bath (HNO_3 15% v v^{-1}) for 24 h. Subsequently, the materials were washed with ultrapure water (resistivity of 18.2 MΩ cm), purified in a PURELAB Ultra system (ELGA, United Kingdom), dried and stored in a place free from contamination. The nickel boats (MILESTONE SRL, Italy) used in the DMA-80 spectrometer were washed with ultrapure water and burned at 650 °C for 3 min in a muffle furnace, as a procedure for Hg decontamination recommended by the manufacturer.

Verification of operational conditions of ICP-MS

The operation conditions of ICP-MS were optimized by verifying the time required for sample cleanup throughout the analysis and possible internal standards (IS) ^{103}Rh, ^{193}Ir and ^{209}Bi.

As reported by Allibone, Fatemian and Walker[1], Hg has the potential to adhere to the introduction sample system, resulting in a memory effect. It was verified the need of the presence of Au in the cleanup solution with the concentration of 1 mg L^{-1} in HNO_3 2% v v^{-1}. The time of cleaning of 60 s between samples was validated through repeated readings of a solution standard containing 5 µg L^{-1} of Hg and and 2% v v^{-1} HNO_3.

The cleaning conditions were also verified for higher Hg concentrations. The concentrations of interest were 20, 30 e 40 µg L^{-1}. For the complete system cleanup (i.e., return of the analytical signal to the 2% v v^{-1} HNO_3 signal), approximately 3 min were required between solutions. In addition to reducing the analytical frequency, 3 min of cleaning time also enhanced the consumption of acid solution with Au. Therefore, the calibration curve was defined from 0.05 to 5.00 µg L^{-1}.

Following the definition of the cleaning parameters for the introduction sample system, the differences among the analytical figures of merit when using ^{103}Rh, ^{193}Ir and ^{209}Bi as IS were verified. Similar sensitivities and determination coefficients (R^2) greater than 0.998 for all calibration curves evaluated.

The accuracy of the calibration curves was assessed through the analyte addition and recovery test, in which a sample solution was spiked with Hg at concentrations of 10 and 100 μg kg^{-1} (in the decomposed solution they are equivalent to 0.1 and 1 μg L^{-1}, respectively). Table S3 indicates that calibration curves with ^{193}Ir or ^{209}Bi may be suitable for achieving satisfactory results in accordance with the AOAC.[2]

Accuracy was also verified using certified reference material (CRM) NIST (National Institute of Standards and Technology) 2702 Marine Sediment. It was determined that the calibration curve using ^{193}Ir (determined value of (430 ± 22) μg kg^{-1}) was in compliance with the certified concentration of (447.4 ± 6.9) μg kg^{-1} of Hg.

Table S3. Analyte recovery of 10 and 100 μg kg^{-1} for the studied analytical curve with isotopes ^{200}Hg and ^{202}Hg.

**Calibration curve**	**Recovery**	
	**Addition of 10 μg kg^{-1}**	**Addition of 100 μg kg^{-1}**
Without IS	51%	69%
With Rh	31%	74%
With Ir	**88%**	**110%**
With Bi	**100%**	**92%**

Adequate recoveries are shown in bold.[2]

Design of Experiments for Hg determination by CV-ICP-OES

Table S4 presents the results of the analysis of variance (ANOVA) conducted on the mathematical model of the CV-ICP-OES system evaluated.

Table S4. ANOVA and validation of the mathematical model for the cold vapor generation system.

	253 nm				194 nm				
	**SS**	**df**	**MS**	**F_{calc}**	**SS**	**df**	**MS**	**F_{calc}**	**F_{tab}**
**Regression**	688549	7	98364	28	192963	7	27566	41	8.9
**Residual**	10398	3	3466		2019	3	673		
**Lack of fit**	7306	1	7306	4.7	1781	1	1781	15	19
**Pure error**	3092	2	1546		238	2	119		
**Total**	619550	10							

Explained variance = 111.14% (253 nm) and 112.37% (194 nm), Maximum explained variance: 99.50% (253 nm) and 99.86% (194 nm). SS: Sum of square, df: Degree of Freedom, MS: Media of square, F_{calc}: Calculated F, F_{tab}: Tabulated F with significance level of 0.05.

Table S5 shows the estimated effects found for the Full Factorial Design 2^3 with central point of the CV-ICP-OES system evaluated.

To determine which effects were significant, it was proposed a null hypothesis (H_0) that the value of effects is confused with their error. With a significance level of 0.05, H_0 is accepted when the *p*-value is greater than the critical *p*-value (0.05). If *p*-value < critical *p*-value, then H_0 is rejected denoting that the effect is significant for the system studied.[3]

Table S5. Values of the estimated effects as well as the effects error for the Full Factorial Design 2^3 with central point.

		Line 253 nm			Line 194 nm		
**Variable**	**Effect**	**Estimated**	**Effect error**	***p*-value**	**Estimated**	**Effect error**	***p*-value**
Rd	1	−2.5	28	0.9366	11.1	7.7	0.2871
Ac	2	−22	28	0.5103	−3.1	7.7	0.7235
Tx	3	581	28	<u>0.0023</u>	309.0	7.7	<u>0.0006</u>
Rd × Ac	12	61	28	0.1589	20.3	7.7	0.1195
Rd × Tx	13	−2.3	28	0.9420	8.5	7.7	0.3863
Ac × Tx	23	−38	28	0.3036	−13.8	7.7	0.2149
Rd × Ac × Tx	123	28	28	0.4229	14.0	7.7	0.2108

Significant values underlined when *p*-value < critical *p*-value (*p*-value = 0.05).

With these considerations, Rd and Ac have non-significant effects. So, in the studied range, these variables do not contribute significantly to a notable increase in the analytical signal and can be fixed within the experimental domain. In order to guarantee that the analytical signal responses were within the experimental domain, Rd was fixed close to the central point, at 7% m v^{-1}. On the other hand, Ac was set at 4% v v^{-1}, aiming to keep the acidity low, both to avoid greater wear of the ICP-OES consumables and to have a lower consumption of reagents.

According to the evaluation of H_0, the only significant effect for the system was Tx for both spectral lines. In this way, a univariate optimization was performed with the Reagent Aspiration Rate, evaluating the sensitivity of the calibration curve and the limits of quantification (instrumental and method). As the Tx effect value was positive, indicating that the higher the value, the greater the analytical signal, Tx of 3 and 4 mL min^{-1} were used in this optimization. Higher aspiration rates were not possible to work with due to the limitations of the cold vapor generation system used.

A hypothesis for this can be explained by the high spectral background value obtained in this region (Figures S2 and S3) and the high values of the calibration linear coefficient at 513 and 400 µg L^{-1}, for aspiration rates of 3 and 4 mL min^{-1}, respectively. The high spectral background can be observed in Figure S2 (a and b) and Figure S3 (a and b) which represents the analytical signal obtained for the CRM NIST 2702 and its respective preparation blank by the ICP-OES software. This background may be associated with the presence of elements with spectral lines adjacent to the 253 nm line, such as Si and Mn, which are present in high concentrations in marine sediment samples. In addition, Si is part of the material composition of the ICP-OES sample introduction system, which may also have contributed to the high spectral background observed, given that this phenomenon was also seen in the preparation blank spectrum (without the presence of the sample). Furthermore, during the decomposition of the samples, Cl_2 may be formed, which has absorption in the same main line of Hg (253 nm). This may also have had a contribution to the low sensitivity in the spectral line of 253 nm.[4]

The Figures S2 to S5 assist in the visualisation of the differences between the 253 and 194 nm lines of Hg in the CV-ICP-OES system.

Figure S2. Analytical signal from the 253 nm line with aspiration rate of 3 mL min^{-1} for (a) preparation blank and (b) CRM NIST 2702.

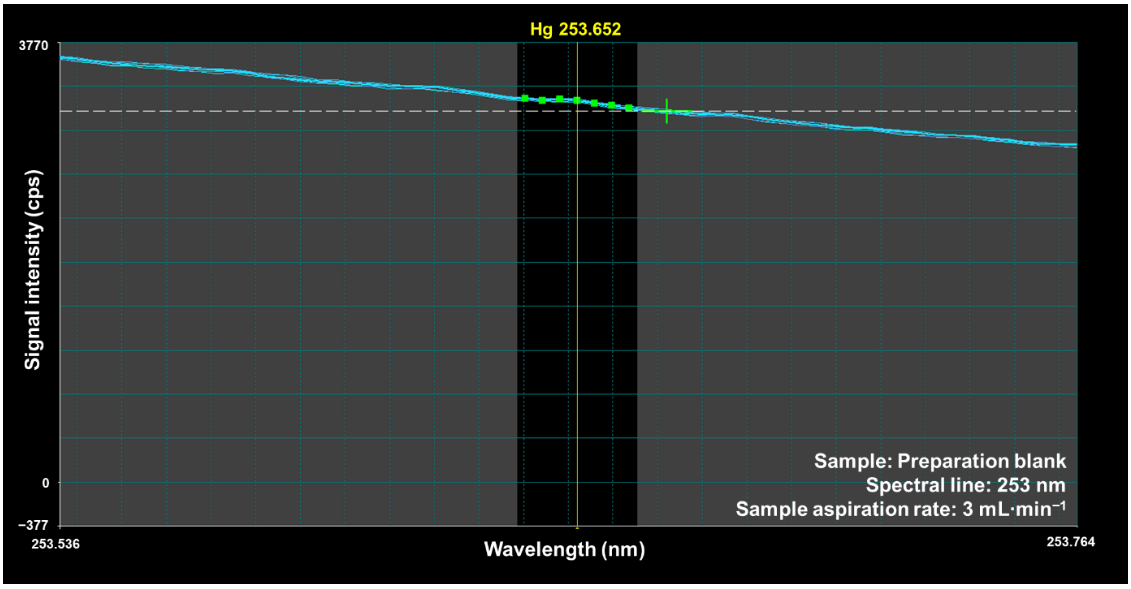

(a)

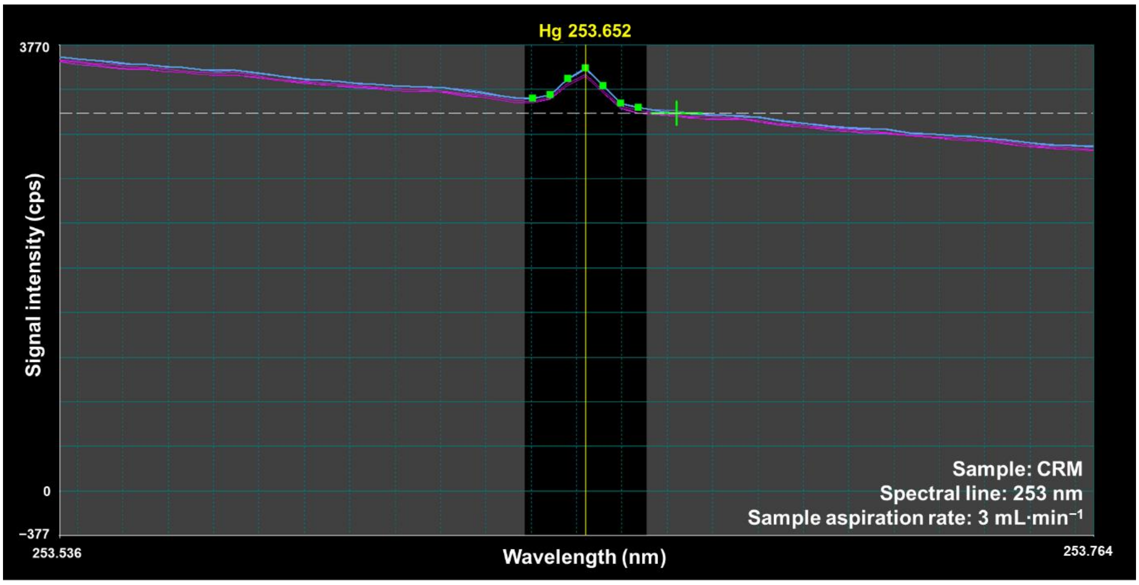

(b)

Figure S3. Analytical signal from the 253 nm line with aspiration rate of 4 mL min^{-1} for (a) preparation blank and (b) CRM NIST 2702.

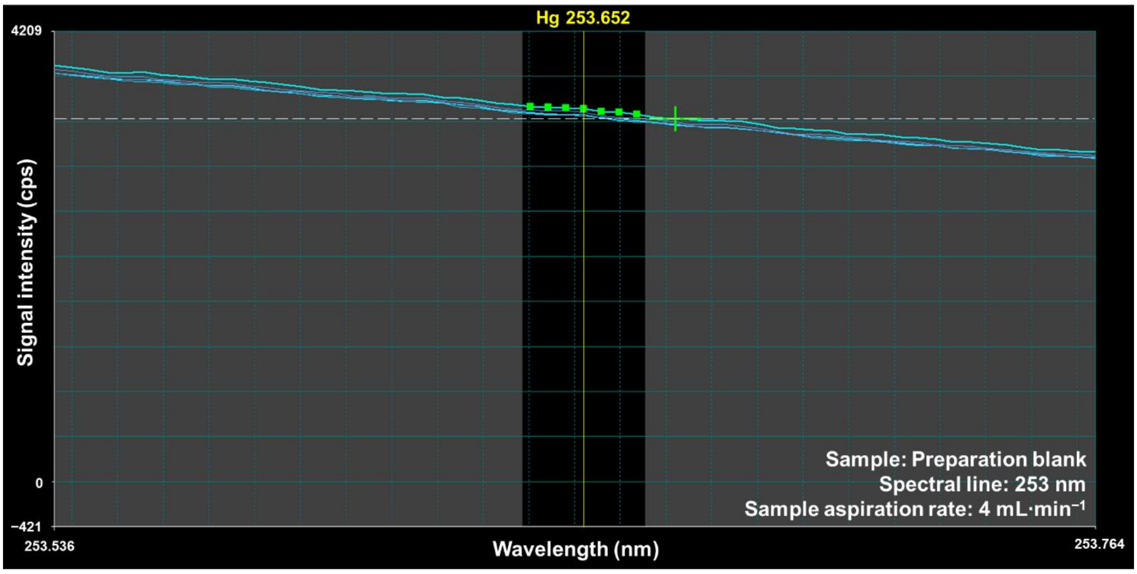

(a)

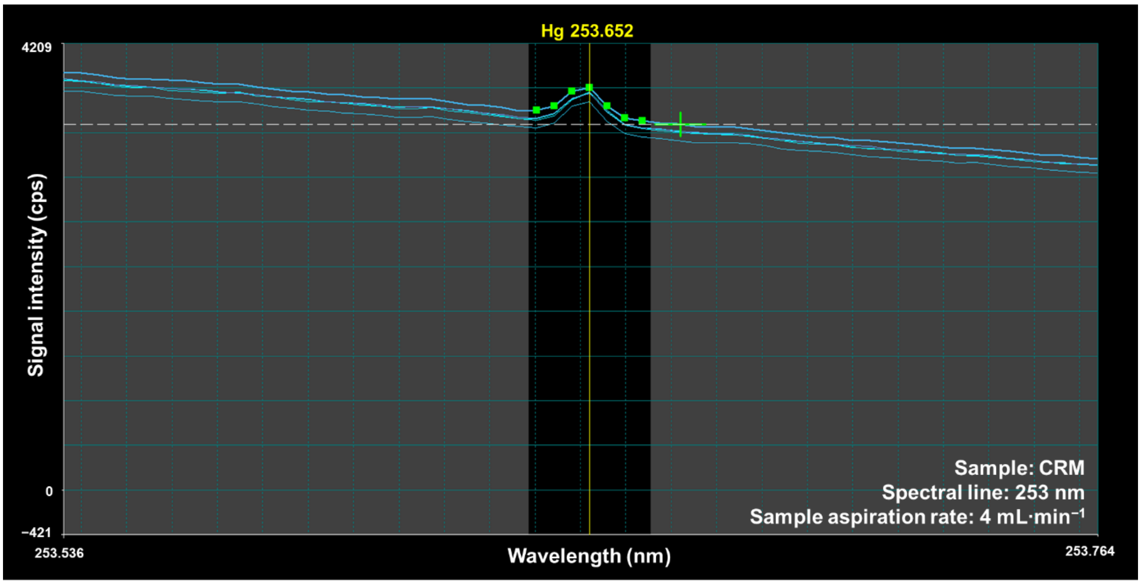

(b)

Figure S4. Analytical signal from the 194 nm line with aspiration rate of 3 mL min^{-1} for (a) preparation blank and (b) CRM NIST 2702.

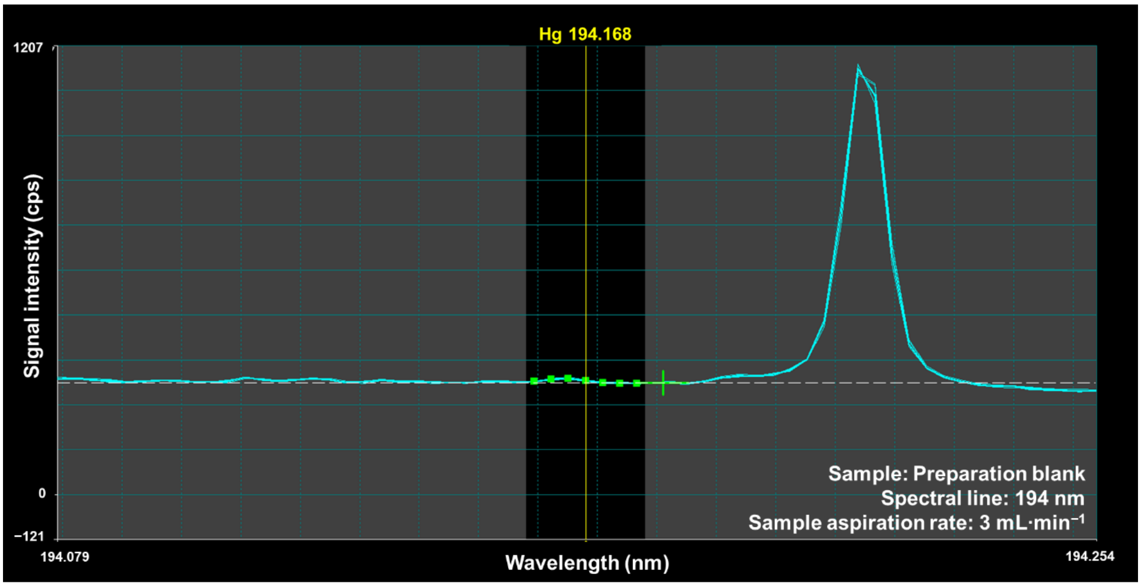

(a)

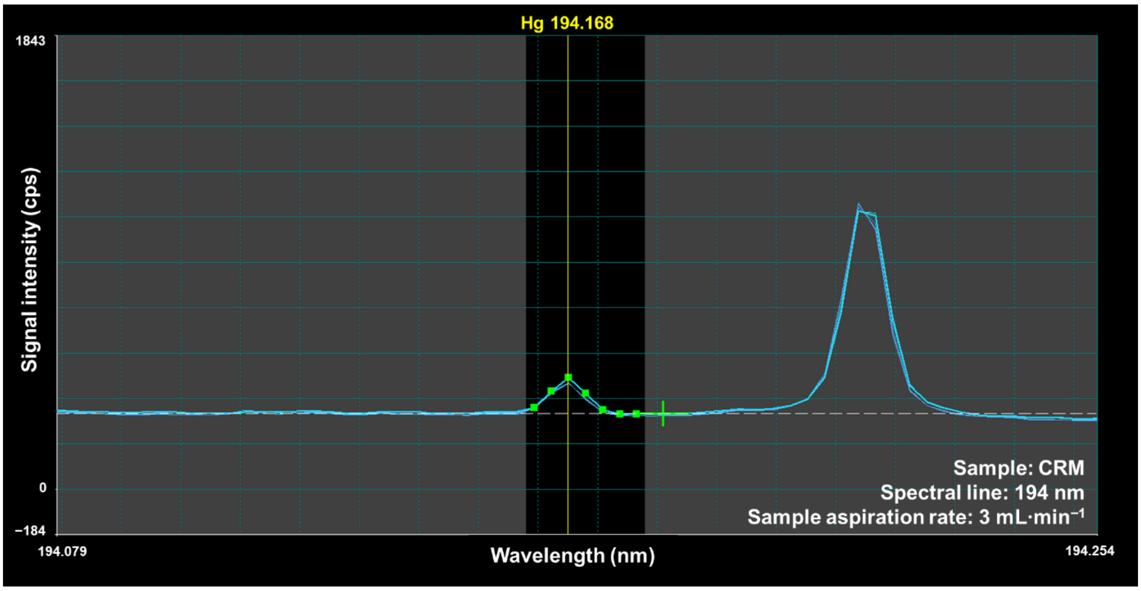

(b)

Figure S5. Analytical signal from the 194 nm line with aspiration rate of 4 mL min^{-1} for (a) preparation blank and (b) CRM NIST 2702.

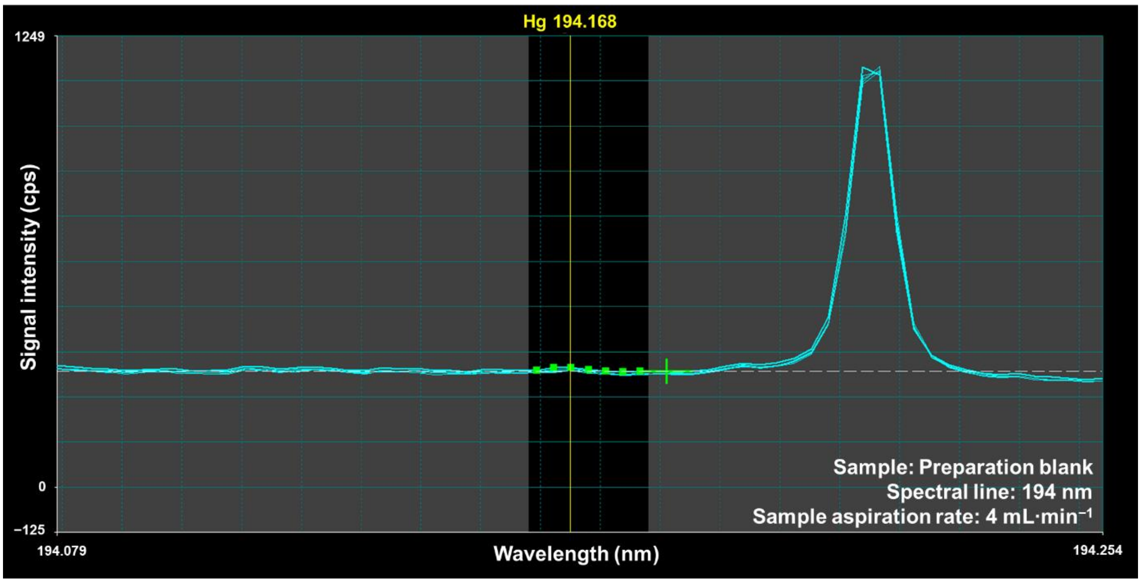

(a)

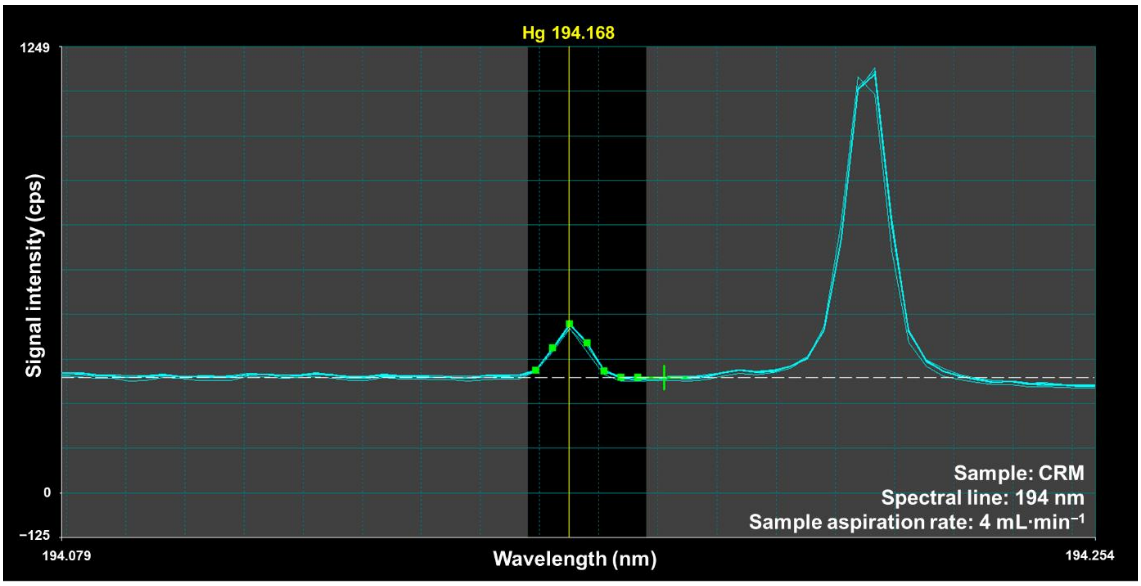

(b)

In Table S6 can be found the values of analyte recovery test for CV-ICP-OES.

Table S6. Analyte recovery of 1 and 5 mg kg^{-1} for the calibration curves studied with lines 253 and 194 nm with sample aspiration rates of 3 and 4 mL min^{-1}.

**Sample Aspiration Rate (mL min^{-1})**	3		4	
**Spectral line (nm)**	253	194	253	194
**1 mg kg^{-1} added analyte**	87%	78%	89%	**96%**
**5 mg kg^{-1} added analyte**	85%	81%	108%	**101%**

Adequate recoveries are shown in bold.[2]

References

(1) Allibone, J.; Fatemian, E.; Walker, P. J. Determination of Mercury in Potable Water by ICP-MS Using Gold as a Stabilising Agent. *J. Anal. At. Spectrom.* **1999**, *14*, 235–239, DOI: 10.1039/a806193i.

(2) *Official Methods of Analysis of AOAC INTERNATIONAL*; Oxford University PressNew York, **2023**, DOI: 10.1093/9780197610145.001.0001.

(3) Teófilo, R. F.; Ferreira, M. M. C. Chemometrics II: spreadsheets for experimental design calculations, a tutorial. *Quim. Nova* **2006**, *29*, 338–350, DOI: 10.1590/s0100-40422006000200026.

(4) UNITED STATES ENVIRONMENTAL PROTECTION AGENCY – US EPA. Method EPA 7471a - Mercury in Solid or Semisolid Waste (Manual Cold-Vapor Technique), **1994**.
